# The Discontent in the British Medical Association

**Published:** 1896-03-28

**Authors:** 


### The Hospital

An Institutional Journal of

The Medical Sciences and Hospital Administration, with

AN EXTRA NURSING SUPPLEMENT.

Vol. XIX.—No. 496.] [March 28, 1896.

### The Discontent in the British Medical Association.

"What does the British Medical Association do for its members?" This is a question asked in the *Medical Magazine* by Dr. Arthur Welsford, of Dover, and Mr. Thomas F. Raven, of Broadstairs, acting as honorary secretaries to a "committee of members of the British Medical Association" interested in the subject of medical defence. The honorary secretaries answer their own question by affirming that it cannot be answered. "It is very difficult," they say, "to frame a suitable reply." They then point out one thing which the Association ought to do for its members, but does not; and they insist that if the Association continues to maintain a *non possumus* attitude on the question of medical defence, there will probably be a "secession from its ranks."

It is not quite just to say, in effect, that the British Medical Association does nothing, or does even very little, for its members. As a matter of fact, it does a great deal. It is sufficient at the present moment to mention the able journal it provides for them at a very moderate cost, the annual congresses it organises in their interests, and the standing committees it selects and guides on behalf of the well-being of the profession as a whole, and of the members of the British Medical Association in particular. If the secretaries of the "Members' Committee" had asked, not what the Association already does, but what more it might yet do and ought to do; and if they had set forth a practical working scheme, embodying the views of a majority of the members of the Association, they would have planted their feet on much firmer ground than that which, with a considerable lack of tactical skill, they have now chosen to occupy. To deny good work which is patent to everybody, and to threaten secession because a little passive resistance is offered to what are admitted to be novel and difficult proposals, is not the way to bring about such a degree of unanimity of feeling as is demanded by the somewhat critical emergency which now confronts all classes of the medical profession.

To be brief, the medical profession, among its other accomplishments, is beginning to take a practical interest in political economy, and to exercise its wits upon questions of values, and the law of supply and demand, and so forth. It subscribes to the British Medical Association an annual income of probably not less than fifteen or sixteen thousand pounds, and the Association makes by its journal a profit of several thousands a year more. A secure income of considerably over twenty thousand a year, its members argue, ought to produce results of a very tangible, practical, and useful kind; and these results ought to be strictly commensurate with the amount of income supplied. The Association has no ornamental objects to serve; it does not exist for purposes of ceremonial or show. It is a practical association, created for practical ends; and it must either fulfil its practical functions at an equitable cost, or be superseded, as other similar organisations have been superseded, by something which will give current money's worth for current money. All this, in general, is incontestable, and in a general way we sympathise with the feelings of those who think the Association might very well do more for its members than it has ever yet done.

But our sympathy with the "progressives" of the British Medical Association would be much deeper if they were more practical, and showed more statesmanship and tactical resource in the devising of means to ends. To take, as an example, this question of medical defence which is now agitating the Association; the "progressives," as we may call them for want of a better name, desire that medical defence, instead of being entrusted, as at present, to two or three relatively insignificant societies, shall be taken up and carried out by all the energy, authority, and prestige of the British Medical Association. That we hold to be a sound position. But where we differ from the progressives is here: they seem to demand that the moment they declare their wish for plums, straightway plums shall fall into their mouths. If that does not come to pass, they threaten to run into a corner and sulk.

Now that is childish. If a certain portion of the British Medical Association desires the Association as a whole to take up the active working of medical defence, then clearly the first duty of the reformers is to convert the whole Association to their views. Instead of this the reformers make application to the paid officials and standing committees of the Association; and when they find these somewhat privileged gentlemen securely planted in easy chairs, and absolutely declining to move, they declare immediately that they will throw up the game. That may be exactly what the officials would like. But could anything be more futile? Could anything be more certain beforehand than that the permanent officials and standing committees would be frightened out of their wits by the prospect of more work, and of a more arduous kind, to do, and of a very decidedly uncertain income to do it with? The reformers should show a little more knowledge of human nature. The permanent officials and standing committees of the British Medical Association are quite happy as they are; all that they wish for is to be left in their armchairs in peace.

What, then, is the practical thing to do? Secede from the Association? Most certainly not. Convert the Association, rouse the Association, utilise the Association. If the officials and committees stand in the way leave them severely alone. Push forward in the direction of least resistance. If the Association as a whole can be converted the work will be done. The officials will then make no more difficulties. Their duty will be to obey, and they will obey. In our judgment the British Medical Association is the only existing organisation in medicine which can carry out the work of medical defence on that high plane and with that efficiency with which it ought to be carried out. It is for those who move on these lines to bring the whole of the thinking portion of the Association round to their views with as little delay as possible.

